# Exploring the potential of augmented reality in rehabilitation: a novel approach for post-stroke unilateral neglect

**DOI:** 10.3389/fspor.2026.1719378

**Published:** 2026-03-02

**Authors:** Ming Yanzhen, Rao Yunhua, Chen Song, Zeng Heqiong, Yang Zufang, Alan Wang

**Affiliations:** 1 School of Artificial Intelligence Academy, Wuhan Technology and Business University, Wuhan, China; 2 Institute of Information and Intelligent Engineering Applications, Wuhan Technology and Business University, Wuhan, Hubei, China; 3 Electronic Information School, Wuhan University, Wuhan, China; 4 AI Research Institute, Langxin Technology Group Co. Ltd., Wuhan, Hubei, China; 5 Auckland Bioengineering Institute, The University of Auckland, Auckland, New Zealand; 6 Medical Imaging Research Center, Faculty of Medical and Health Sciences, The University of Auckland, Auckland, New Zealand; 7 Centre for Brain Research, The University of Auckland, Auckland, New Zealand; 8 Centre for Co-Created Ageing Research, The University of Auckland, Auckland, New Zealand

**Keywords:** augmented reality, neural network, NeuroNavAR, post-stroke, segmentation, unilateral neglect

## Abstract

**Purpose:**

Unilateral neglect affects up to 80% of right hemisphere stroke survivors and poses a significant barrier to rehabilitation. It is a strong predictor of poor prognosis and leads to prolonged hospital stays, yet no established treatment currently exists.

**Methods:**

We propose an innovative approach, NeuroNavAR, a novel treatment method that utilizes neural network models to train the human nervous system under the guidance of augmented reality (AR). This method focuses on an AR-enhanced smart training program specifically for post-stroke spatial neglect. While the current implementation runs on a GPU server for technical validation, the application is designed for future deployment on Android and iOS devices. It employs image processing algorithms to identify real-world objects, such as clocks and chairs, segment their contours, and generate virtual bee patterns. These virtual bees navigate along the contours of the objects, and patients are encouraged to follow the bee patterns, receiving rewards upon completing a cycle. If patients fail to complete the cycle, virtual birds are introduced into their field of view to guide them back to the bee patterns, thereby enhancing engagement.

**Results:**

This study reports only on the technical feasibility of the system. No clinical, behavioral, or usability data were collected. On a test dataset comprising 537 indoor images from the ADE20K dataset (accessed June 2024), the segmentation achieved a mean Intersection over Union (IoU) of 76.70% (SD = 8.2%) and an accuracy of 88.51% (SD = 4.7%).

**Conclusion:**

The NeuroNavAR program represents a preclinical engineering feasibility study that provides a more intuitive and user-friendly training experience compared to traditional rehabilitation methods. Its innovative use of augmented reality and real-time feedback is designed to support, but has not yet demonstrated, improved rehabilitation outcomes for patients with unilateral spatial neglect. Clinical validation is planned for 2026.

## 1 Introduction

**Figure 1:**
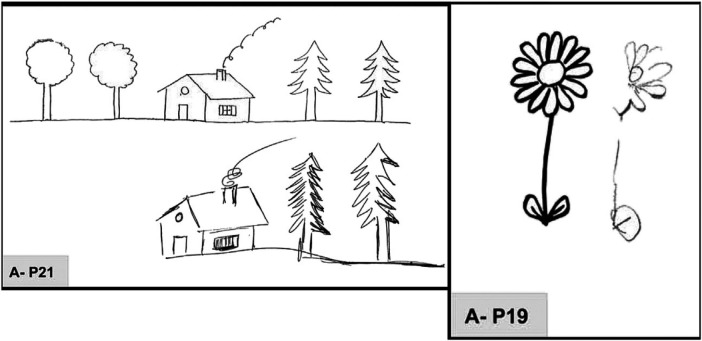
Drawing by copy. P19, copy of a daisy: omission of left petals, right-sided perseveration in the lower petals. P21, copy of a multiple object drawing: omission of two left-sided trees, perseveration on the house (chimney, and window), and on the right-hand sides of the two right-sided pine trees [4].

Patients undergoing stroke rehabilitation frequently experience visuospatial neglect (VSN) [1], a condition characterized by deficits in processing visual information, which affects their perception of object positions and spatial relationships. One of the primary symptoms of this condition is unilateral neglect, also known as unilateral spatial neglect (USN) [2, 3]. This attentional disorder arises when a stroke impairs the lateral fissure network, comprising the superior/middle temporal cortex, parietal cortex, and ventrolateral prefrontal cortex, resulting in the brain’s inability to properly process spatial information. As shown in Figure 1, USN patients often fail to respond to stimuli on one side, typically the left side due to right hemisphere lesions. This results in significant disruptions to daily activities, such as perceiving only the right side of objects while walking, dressing, or eating, which increases the risk of disabilities or falls. Consequently, patients face prolonged hospital stays and elevated psychological and economic burdens. Therefore, in-depth research into this phenomenon is crucial for improving rehabilitation strategies post-stroke.

The complexity of USN renders both its diagnosis and treatment challenging. Current international clinical guidelines from the European Stroke Organisation (ESO, 2021) and the Cochrane Collaboration (2023) identify *visual scanning training* (VST) as the first-line non-pharmacological intervention for post-stroke neglect, supported by moderate-certainty evidence. Crucially, both guidelines emphasize that *effective* VST must involve active, goal-directed exploratory actions—specifically, coordinated rotations of the eyes, head, and trunk toward the neglected side [5]. This multimodal motor engagement is essential for stimulating the impaired dorsal (top-down) and ventral (bottom-up) attention networks [6], thereby fostering cortical reorganization and functional neuroplasticity—the biological basis for long-term recovery [7].

Despite this consensus, conventional VST delivery—often via paper-and-pencil tasks, computer screens, or therapist-guided exercises—suffers from limited ecological validity, poor patient engagement, and insufficient emphasis on full-body exploratory actions in real-world contexts. Emerging technologies offer promising alternatives: prism adaptation (PA) induces sensorimotor recalibration but lacks generalizability beyond the trained task [8]; immersive virtual reality (VR) enhances engagement yet may cause cybersickness and isolates users from their physical environment [9].

To bridge this gap, we propose *NeuroNavAR*, an Augmented Reality (AR)-enhanced intelligent rehabilitation platform explicitly designed to operationalize the ESO/Cochrane-endorsed principles of explorative action training. Our system leverages real-time object detection and segmentation to identify everyday indoor objects—such as clocks, chairs, and tables—via a mobile device camera. Patients interact with a gamified interface featuring a virtual *bees* that traverses the contours of detected objects; its movement is deliberately slowed on the neglected side to encourage sustained contralesional attention. Successful completion of a contour path yields immediate rewards, while missed tracking triggers guiding *birds* to gently redirect focus—embodying principles of errorless learning and intrinsic motivation. Most critically, the AR overlay is spatially anchored to real-world objects, thereby requiring patients to dynamically rotate their eyes, head, and trunk to follow the virtual agents within their natural environment. This embodied interaction is designed to simultaneously engage the dorsal network (through voluntary, goal-driven search planning) and the ventral network (through salient, moving visual stimuli), directly targeting the core pathophysiology of USN.

Furthermore, NeuroNavAR is conceived not merely as a therapeutic tool but as a multimodal research platform. As outlined in our broader project framework, it will be integrated with concurrent EEG and MRI data collection to investigate training-induced neural plasticity and to develop predictive models of individual treatment response—paving the way for truly personalized, AI-driven neurorehabilitation. The present paper reports the technical feasibility and validation of the core AR pipeline—including object recognition, contour generation, and real-time AR rendering—laying the essential groundwork for upcoming clinical trials with stroke survivors in New Zealand and China (Wuhan) in Year 2.

## 2 Methodology

### 2.1 System architecture

Augmented Reality (AR) presents a promising approach to enhance motivation in post-stroke rehabilitation training. AR involves the process of integrating computer-generated virtual elements into the real world. Using cameras on electronic devices such as tablets, these virtual images and graphics augment the real-world view. This virtual augmentation happens in real-time, effectively blending the real with the virtual. In this study, we explore how AR can serve as a visual scanning therapy for patients with Visuospatial Neglect (VSN), thereby enhancing their engagement and independence during rehabilitation.

**Figure 2:**
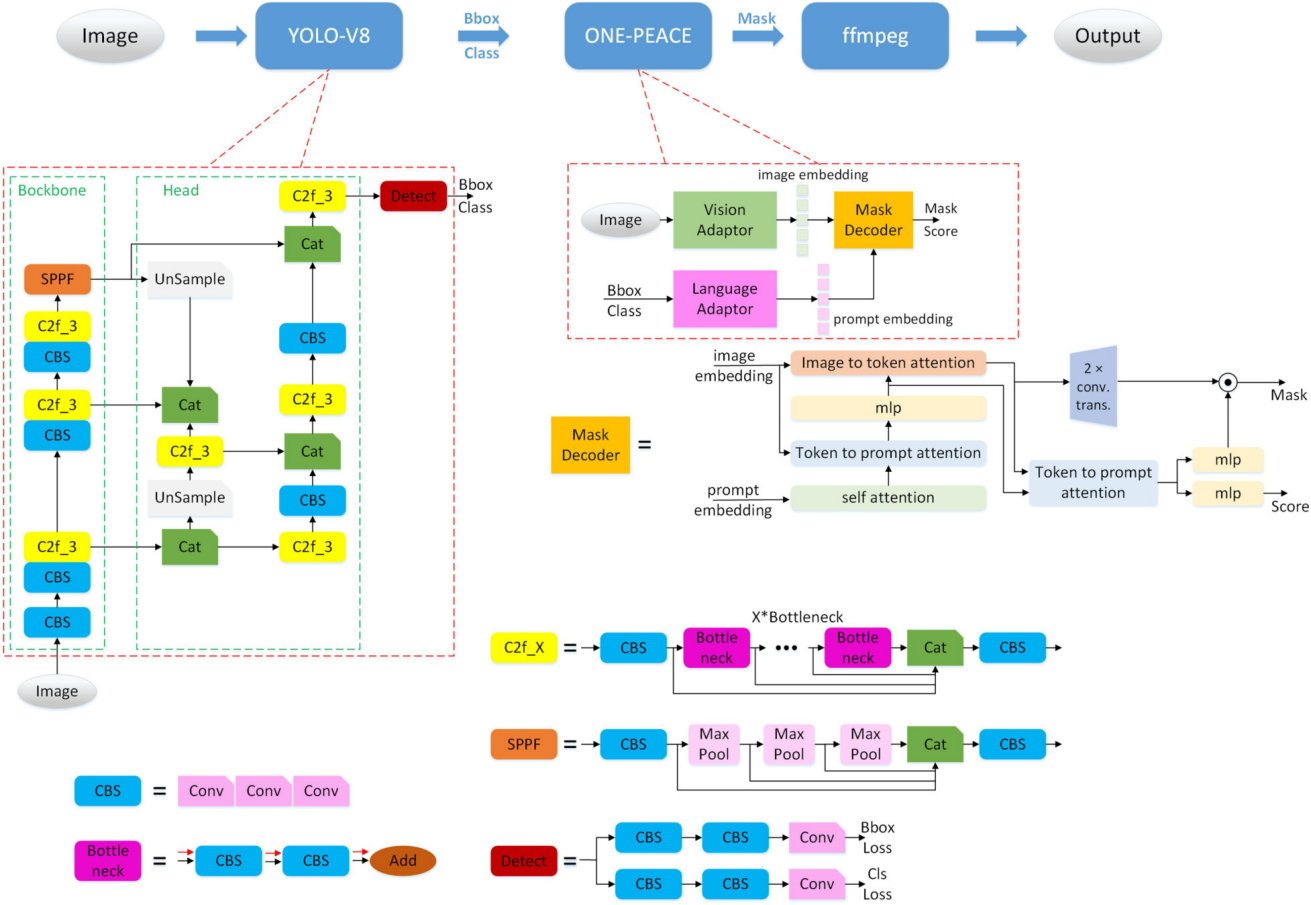
System architecture.

As illustrated in Figure 2, the system architecture begins with input images processed by the YOLO-V8 detection network [10], which identifies objects such as tables and chairs. These objects are then input into the ONE-PEACE segmentation network [11], which produces masks to obtain edge information of the objects. Finally, FFmpeg is used to overlay virtual bee stickers along the object’s edges. These virtual bees circulate the object’s boundary, rotating more slowly and performing more cycles on the patient’s neglected side to enhance attention to that area. Upon completing a full cycle, the program rewards the patient. If the patient fails to locate the virtual bees, a virtual bird appears to guide the patient back to the bees’ location.

Both models are used in their official pre-trained configurations without fine-tuning. Specifically, we employ the Ultralytics YOLOv8s model (yolov8s.pt), pre-trained on the COCO dataset with an input resolution of 640×640. For segmentation, we use the official ViT-H/14 checkpoint from Alibaba’s ONE-PEACE release (onepeace_seg_cocostuff2ade20k.pth), with the vision encoder frozen during inference.

### 2.2 Implementation and runtime details

All experiments were conducted on a server equipped with one NVIDIA V100 GPU (32 GB memory) and 16 vCPUs (Intel(R) Xeon(R) Gold 6,130 CPU @ 2.10 GHz). The software environment includes Ubuntu 18.04, CUDA 12.1, PyTorch 2.1.1, and FFmpeg 4.4.4.

Inference timing was measured end-to-end on the V100 GPU: YOLOv8 detection takes approximately 10 ms per image, ONE-PEACE segmentation requires 260 ms, and contour rendering via FFmpeg adds 50 ms. The full pipeline thus achieves a throughput of approximately 3 frames per second (FPS), with an average latency of 320 ms per frame.

To ensure therapeutic relevance, only objects whose bounding box occupies more than 0.2% of the total image area are considered. Among these, we retain the top-5 largest objects as “significant” targets for AR interaction, filtering out distant or irrelevant background items. This area threshold (0.002 relative to image size) was empirically determined to balance detection reliability and clinical relevance in typical indoor rehabilitation settings.

### 2.3 Model network architecture

This section provides a detailed description of the architectural components of the YOLO-V8 and ONE-PEACE networks, highlighting the primary functions and unique features of each component.

#### 2.3.1 YOLO-V8 network architecture

The YOLO-V8 network is composed of three main components: the Backbone, the Head, and the Detect module.

**Backbone:** The backbone is the core network responsible for progressively extracting image features. It is an improved version of traditional classification networks, optimized for capturing detailed visual information.

**Head:** The head is designed to integrate features from various stages of the network, facilitating the fusion of information to provide richer feature representations.

**Detect:** The detect module generates detection boxes and classifies objects within those boxes, which form the final output for object detection tasks.

The network includes several specialized modules to enhance its performance:

**CBS:** This module consists of three integrated convolutional layers, as described in [12], and serves as a fundamental building block for feature extraction.

**Bottleneck:** Built on top of two CBS modules, the bottleneck structure [13] condenses feature maps into more compact and informative representations.

**C2f_X:** This module extends the bottleneck by incorporating skip connection strategies [13], which facilitate efficient feature propagation.

**SPPF:** Based on Max Pool downsampling, this module also uses skip connections to ensure the extraction of features at multiple levels of granularity.

In addition to these modules, YOLO-V8 utilizes spatial operations like Upsample (upsampling) [14] and Max Pool (max pooling) [15] to manipulate image data efficiently. Max pooling assists in feature extraction and dimensionality reduction, whereas upsampling is crucial for reconstructing or enhancing image resolution.

#### 2.3.2 ONE-PEACE network architecture

The ONE-PEACE network consists of three primary components: the Vision Adaptor, the Language Adaptor, and the Mask Decoder.

**Vision adaptor:** This component encodes visual inputs, such as images, into feature representations known as image embeddings, which capture essential visual characteristics.

**Language adaptor:** It encodes textual information from detection boxes and class labels into prompt embeddings, encapsulating semantic details.

**Mask decoder:** As detailed in [16], the mask decoder integrates the image and prompt embeddings through feature fusion, resulting in a mask. This process employs numerous self-attention modules [17].

**Image to Token Attention and Token to Prompt Attention:** These are specialized self-attention mechanisms where the input extends from one to two variables, enabling complex interaction modeling.
**MLP:** A multi-layer perceptron (MLP) [18] acts as a fully connected network to further process these embeddings, contributing to the final output.

Through this sophisticated design, the ONE-PEACE network effectively integrates vision and language modalities, enabling comprehensive feature extraction and synthesis for advanced object detection and segmentation tasks.

#### 2.3.3 Mask2Former baseline

To validate the design choice of our two-stage pipeline (YOLOv8 + ONE-PEACE), we include a comparison with Mask2Former [19], a state-of-the-art universal segmentation architecture. Specifically, we use the official Swin-Large variant trained on ADE20K with input resolution 384×384 (mask2former_swin_large_384_ade20k.pth). No fine-tuning was performed; inference was run directly on the 537 test images using the original image resolutions. This setup allows a fair comparison under identical data and evaluation protocol.

### 2.4 Processing steps

The pseudocode for the experimental procedure is presented as algorithm 1. The algorithm consists of two nested loops. The outer loop iterates over the images in the test dataset, while the inner loop processes each bounding box (bbox) detected within an image. These bounding boxes are obtained using the YOLO-v8 model. The steps within the inner loop are as follows: first, the ONE-PEACE model is used to segment the object within the bbox, resulting in the delineation of the object’s edges. Subsequently, a distance filter is applied to exclude objects that are too far from the patient, as these would not be effective for rehabilitation training.

In practice, this filter is implemented by selecting only those objects whose bounding box area exceeds 0.2% of the image and retaining the top-5 by area.

Only objects within the therapeutic distance range are retained for further processing. Finally, the mask obtained from segmentation is converted into a contour representation. After the inner loop completes, the FFmpeg tool is employed to overlay these contours onto the image, along with class information and the virtual bee animations, ultimately saving the result as a GIF file.

Algorithm 1 **Processing steps.**

1: **for** each image *img* in *test_loader* **do**

2:  *bboxes*, *class_names* ← YOLO-v8(*img*)

3:  **for** each *bbox* in *bboxes* **do**

4:   *mask* ← ONE-PEACE(*bbox, img*)

5:   *mask* ← filterDistantObjects(*mask,bbox,img,distance_threshold*)

6:   *contour* ← mask2contour(*mask*)

7:  **end for**

8:  *gif* ← FFmpeg(*contours, class_names, img*)

9: **end for**

## 3 Experiments

### 3.1 Dataset

To validate the feasibility of our proposed method, we selected indoor scenes from the validation set of the ADE20K dataset [20] as our test set. ADE20K provides extensive annotations of scenes, objects, and object parts. It contains 25,000 images of complex everyday scenes, featuring a wide variety of objects within natural spatial environments. On average, each image contains 19.5 instances and 10.5 object classes. For our test dataset, we selected a total of 537 images, specifically focusing on indoor scenes comprising 50 labels corresponding to object categories found in such environments.

### 3.2 Experimental setup

In this study, the experiments were conducted using an NVIDIA V100 GPU with a memory capacity of 32 GB. The inference server was running on Ubuntu 18.04, with CUDA version 12.1. The software framework used was PyTorch version 2.1.1, and for media processing, FFmpeg version 4.4.4 was utilized.

During the inference process for detection and segmentation models, the batch sizes were set to 32 and 8, respectively. Both image embedding and prompt embedding were configured with a length of 1024. To achieve model size reduction and inference acceleration, the precision of the models was reduced from float32 to float16.

We evaluated the impact of this mixed-precision inference and observed a negligible performance drop: IoU decreased by only 0.2% (from 76.90% to 76.70%) and Accuracy by 0.1% (from 88.61% to 88.51%). This minor degradation is acceptable given the substantial reductions in memory usage and inference latency, which are critical for future mobile deployment.

### 3.3 Evaluation metrics

In segmentation tasks, Accuracy [21] and Intersection over Union (IoU) [22] are commonly used evaluation metrics, each emphasizing different aspects of performance.

#### 3.3.1 Accuracy

Accuracy represents the proportion of correctly classified pixels to the total number of pixels. It is calculated as Equation 1:

$$\text{Accuracy}=\frac{\text{TP}+\text{TN}}{\text{TP}+\text{TN}+\text{FP}+\text{FN}}$$ (1)

where TP is true positives, TN is true negatives, FP is false positives, and FN is false negatives. Accuracy is suitable for situations with minor class imbalance, but it can be misleading in cases of severe class imbalance.

#### 3.3.2 Intersection over union (IoU)

IoU is a metric specifically designed for segmentation tasks, measuring the overlap between the predicted area and the ground truth area. It is calculated as Equation 2:

$$\text{IoU}=\frac{\text{Area of Overlap}}{\text{Area of Union}}=\frac{\text{TP}}{\text{TP}+\text{FP}+\text{FN}}$$ (2)

The IoU value ranges between 0 and 1, with 1 indicating perfect overlap. It is more suitable for evaluating segmentation performance, especially in scenarios with class imbalance, as it focuses more on the accuracy of the predicted regions.

In summary, Accuracy provides an overall performance metric, while IoU emphasizes the quality of segmentation.

### 3.4 Baseline comparison

To justify our two-stage pipeline, we compared its performance against Mask2Former [19], a powerful end-to-end segmentation model. Both methods were evaluated on the same 537-image indoor subset of ADE20K, using per-class mean IoU and Accuracy computed over all pixels of each class across the dataset. No post-processing or fine-tuning was applied to either method to ensure a fair comparison.

## 4 Results

### 4.1 Per-class segmentation performance

**Figure 3:**
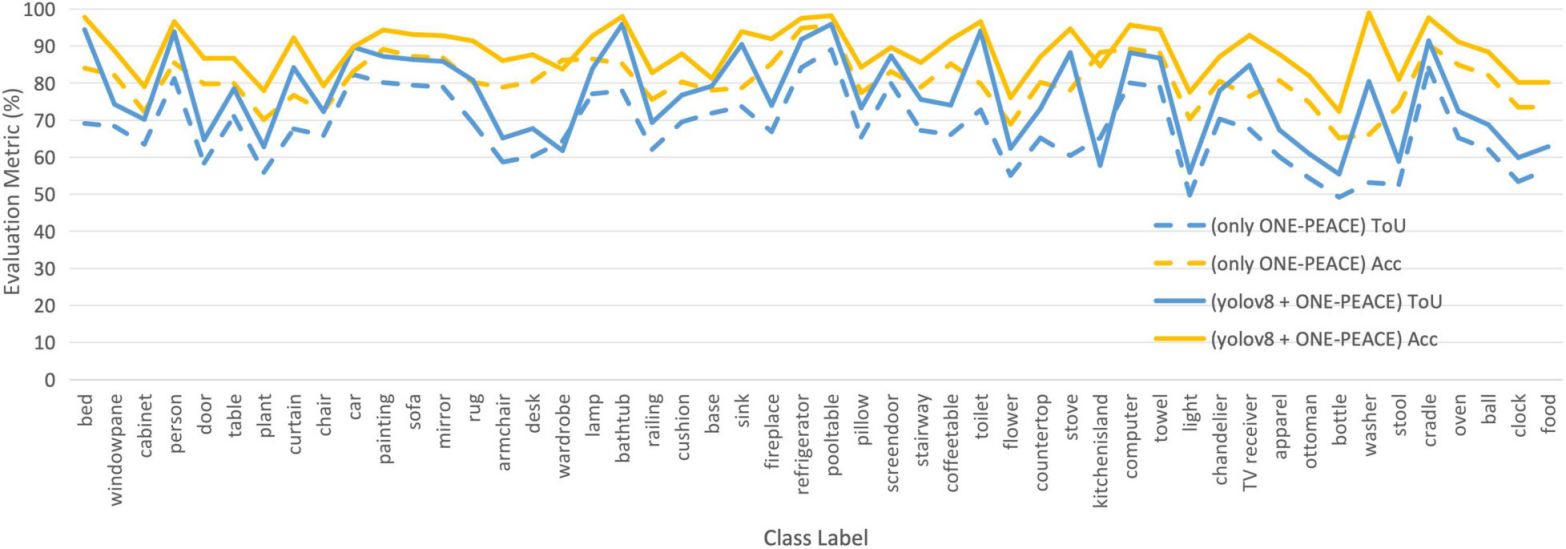
Per-class segmentation performance comparison on the 537-image test set. The *x*-axis lists the 50 common indoor object categories (e.g., bed, chair, lamp), and the *y*-axis shows the corresponding Intersection over Union (IoU) and Accuracy (Acc) values in percentage for both our pipeline (YOLOv8 + ONE-PEACE, solid line) and Mask2Former (dashed line).

As illustrated in Figure 3, we present the per-class segmentation performance of both our proposed pipeline (YOLOv8 + ONE-PEACE) and the Mask2Former baseline.

The average Intersection over Union (IoU) and accuracy (Acc) of our pipeline are 76.70% and 88.51%, respectively. In comparison, Mask2Former achieves 65.88% IoU and 80.02% accuracy on the same test set. The IoU values for our method range from 55% to 95%, while those for Mask2Former range from 49.21% to 89.01%. Notably, our method consistently outperforms Mask2Former across most categories, especially on large, structurally salient objects such as *bed* (94.43% vs. 69.12%), *lamp* (95.91% vs. 77.88%), and *fireplace* (91.80% vs. 84.20%). This demonstrates the advantage of our two-stage approach in accurately segmenting clinically relevant indoor objects for AR-based therapy.

### 4.2 Statistical summary of per-class performance

To provide a more comprehensive assessment of segmentation reliability across diverse object categories, we report descriptive statistics of the per-class Intersection over Union (IoU) and Accuracy on the 537-image test set (comprising 50 indoor object classes). As shown in Table 1, the mean IoU is 76.70% (SD = 11.2%), with a 95% confidence interval of [73.5%, 79.9%], while the mean Accuracy is 88.51% (SD = 6.8%), with a 95% CI of [86.5%, 90.5%]. The standard deviation reflects notable inter-class variability—e.g., high-performing classes like *lamp* (IoU = 95.91%) and *bed* (IoU = 94.43%) contrast with lower-performing ones such as *stove* (IoU = 57.80%) and *bottle* (IoU = 55.45%). This variability underscores the challenge of generalizing segmentation to small, occluded, or low-texture objects, which we discuss in Section 5.

**Table 1:** Descriptive statistics of per-class segmentation performance (50 object categories).

Metric	Mean (%)	Std Dev (%)	Min (%)	Max (%)	95% CI (%)
IoU (Ours)	76.70	11.2	55.45	95.91	[73.5, 79.9]
Acc (Ours)	88.51	6.8	72.37	98.93	[86.5, 90.5]
IoU (Mask2Former)	65.88	10.6	49.21	89.01	[62.8, 68.9]
Acc (Mask2Former)	80.02	7.1	65.20	95.60	[78.0, 82.0]

These statistics confirm that while average performance is strong, the tail distribution (lower-performing classes) may impact AR interaction fidelity in real-world scenarios. Future work will prioritize robustness for these critical categories.

### 4.3 Visualization

**Figure 4:**
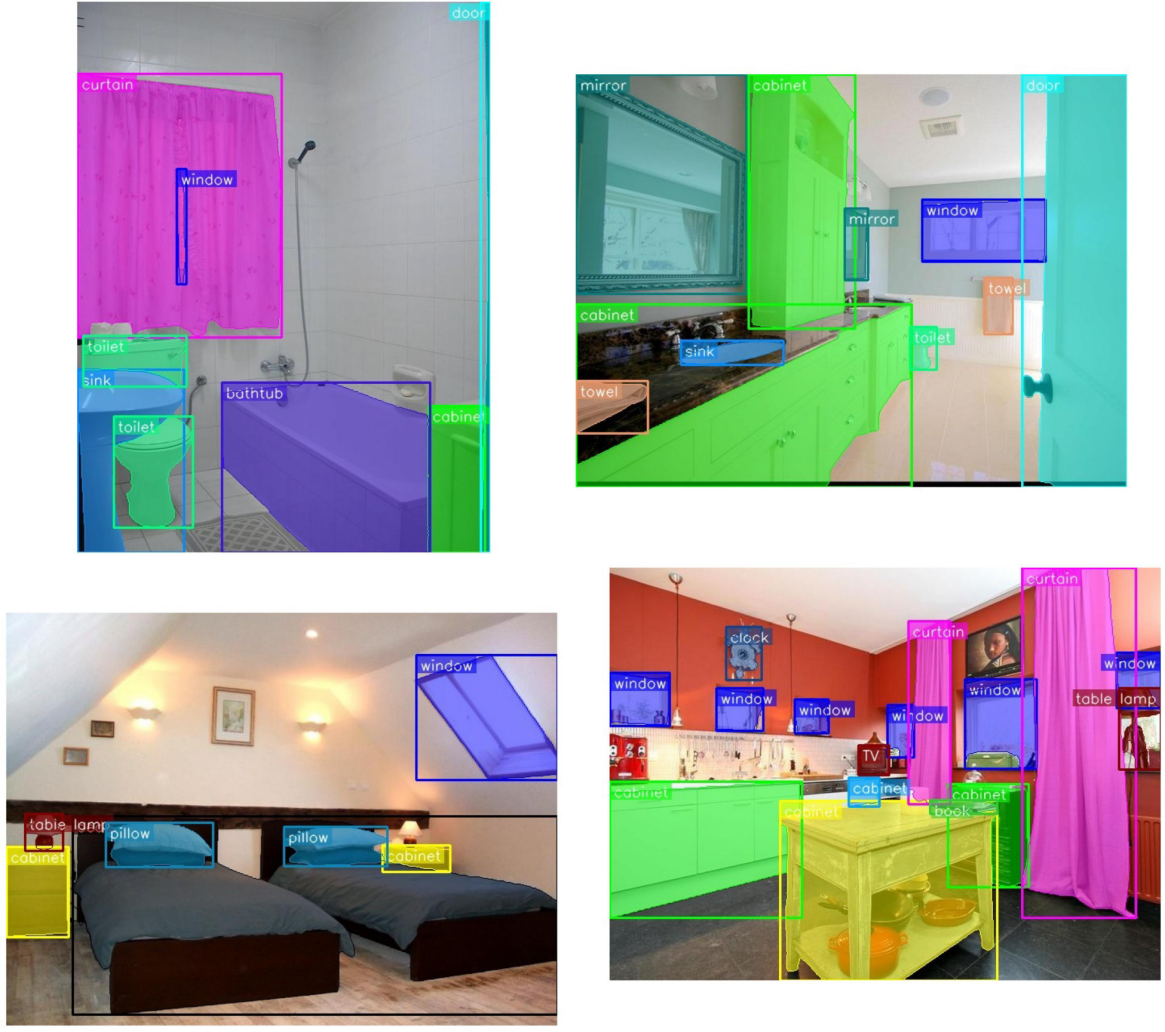
Combined visualization of object detection and segmentation results. Bounding boxes (solid rectangles) from YOLO-v8 indicate detected object locations, with class labels shown at the top-left corner of each box. Semi-transparent colored overlays represent segmentation masks generated by the ONE-PEACE model, precisely delineating object boundaries. Different colors correspond to distinct object instances, ensuring clear visual separation and annotation consistency.

Figure 4 presents the combined visualization of object detection and segmentation results. In these images, detected objects are highlighted with bounding boxes, while their precise boundaries are delineated with contours. The detected category names are displayed in text adjacent to each object. This visualization demonstrates the accuracy of our system in identifying and precisely segmenting various indoor objects such as chairs, tables, and bed.

**Figure 5:**
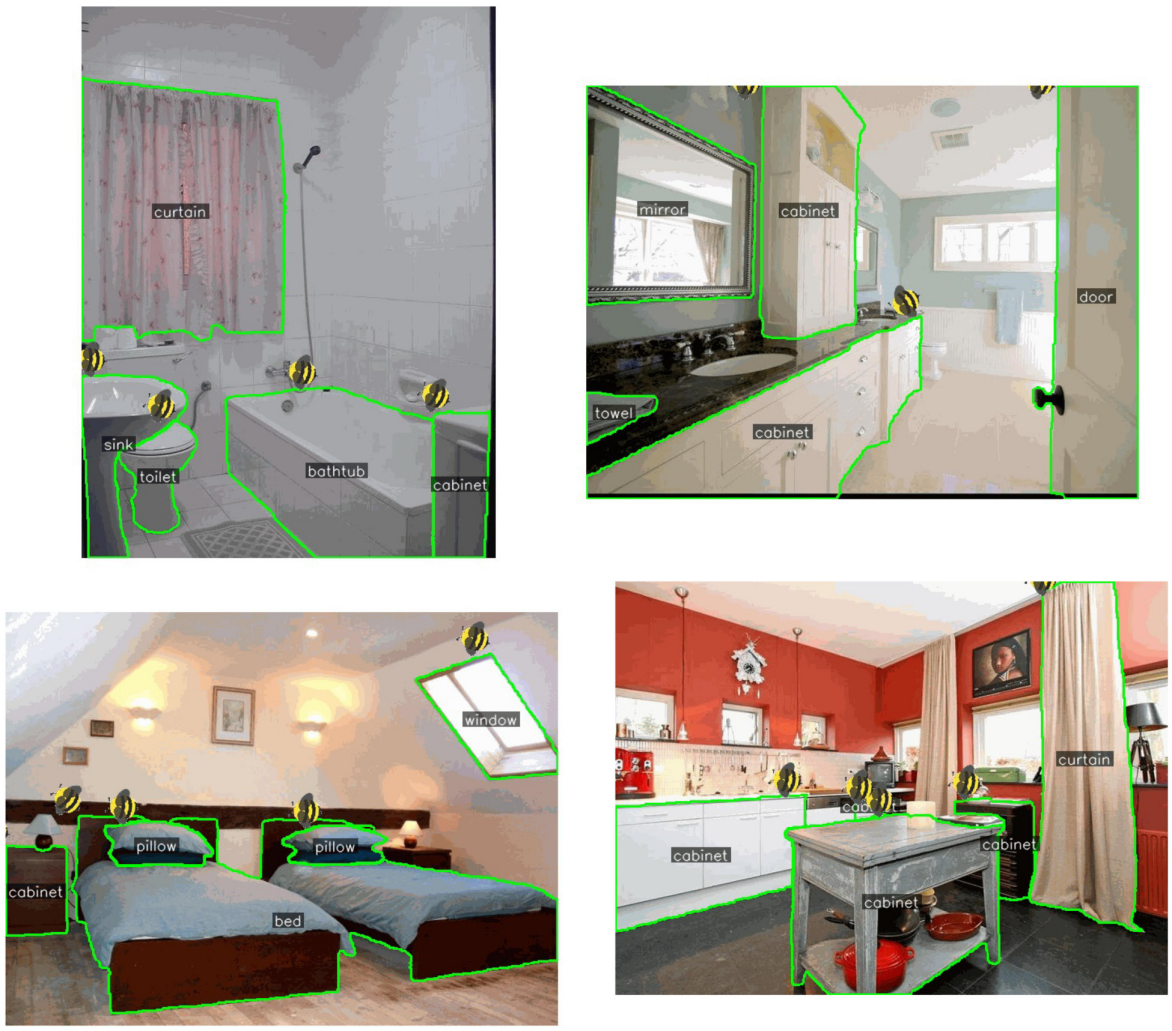
Final augmented reality (AR) output of the NeuroNavAR system. Virtual bees (yellow icons) animate along the segmented object contours (derived from Figure 4), with slower movement on the patient’s neglected side (typically the left) to encourage attentional redirection.

Building upon this detection and segmentation foundation, Figure 5 shows the final augmented reality output where virtual bees move along the contours of the segmented objects. The bees move more slowly on the neglected side of the patient (typically the left side for right hemisphere stroke survivors) to encourage attention to that area. When patients successfully follow the complete path, they receive visual rewards. If patients fail to complete the cycle, virtual birds appear to guide their attention back to the bee patterns.

This two-stage visualization—first demonstrating accurate object detection and segmentation, then showing the therapeutic application—illustrates how our system transforms raw image data into an engaging rehabilitation tool. The accurate detection and segmentation of visible indoor objects provide comprehensive contour information, which, combined with the animated bees and real-time feedback mechanisms, offers an effective method for addressing post-stroke unilateral neglect. Compared to previous treatment methods, this approach offers a more automated, contextually relevant, and engaging solution that can be easily deployed in various environments, ultimately proving to be more intuitive and effective for patient rehabilitation.

## 5 Discussion

The experimental results demonstrate that deep learning-based object segmentation algorithms achieve high accuracy in indoor image segmentation tasks, with a mean IoU of 76.70% and accuracy of 88.51%. This provides a solid technical foundation for developing a novel augmented reality (AR) approach to address post-stroke unilateral spatial neglect (USN).

Our study focuses on integrating intelligent image processing with AR-based game design to create a rehabilitation application for USN patients. A critical innovation lies in the system’s ability to accurately recognize everyday objects (e.g., clocks, chairs) in natural environments and delineate their contours in real time, enabling ecologically valid training that bridges the gap between clinic and daily life.

Compared to existing interventions—such as prism adaptation, non-ecological visual scanning tasks, or immersive VR—our AR approach offers enhanced ecological validity, accessibility, and engagement. By embedding gamified elements directly into the patient’s real-world view, the NeuroNavAR system promotes active visual exploration of the neglected hemifield, encouraging dynamic contralesional rotations of the eyes, head, and trunk—key components of effective exploratory action training.

### 5.1 Safety and risk mitigation

To minimize risks such as cognitive overload or visual fatigue, the system incorporates: (1) simple, non-cluttered virtual stimuli (a moving bee and guiding birds); (2) session duration limits (<20 min); (3) object-anchored (not head-fixed) AR to reduce motion discomfort; and (4) adjustable difficulty levels. Future versions will include real-time engagement monitoring with automatic pausing.

Importantly, our comparison with Mask2Former—a leading end-to-end segmentation model—demonstrates that the proposed two-stage pipeline (YOLOv8 + ONE-PEACE) achieves significantly higher segmentation accuracy on clinically relevant indoor objects. This validates our design choice: decoupling detection and segmentation allows the system to focus computational resources on salient, large objects that are most useful for AR therapy, whereas end-to-end models like Mask2Former distribute attention more uniformly, sometimes compromising performance on key categories.

Despite these strengths, limitations remain. First, our experiments were conducted on static images using a GPU server; real-time, on-device performance on Android or iOS has not yet been validated. The current work constitutes a technical feasibility study; a mobile deployment prototype using TensorFlow Lite and ONNX Runtime is under active development to enable on-device inference on consumer smartphones. Optimization using lightweight architectures [e.g., MobileSAM [23]] is therefore essential for seamless mobile deployment. Additionally, as a camera-based AR application intended for future clinical and home use, considerations regarding privacy and data protection—including secure handling of visual data, user consent protocols, and compliance with regulations such as HIPAA and GDPR—will require careful attention in subsequent development phases.

While average segmentation quality is high, the observed inter-class variability (SD of IoU = 11.2%) suggests that object-dependent performance must be considered in clinical deployment—particularly for small or partially occluded items commonly encountered in home environments.

Second, and critically, no human-subject testing has been conducted at this stage. Consequently, we lack empirical data on usability metrics such as AR sequence completion time, task success rate, or user satisfaction. This is consistent with our project’s phased development plan: **Year 1** focuses on core system engineering and algorithm integration, while **Year 2** is dedicated to clinical validation.

To address this gap, we have planned a pilot clinical trial in Year 2 across sites in New Zealand and China (Wuhan), involving 25 stroke survivors with chronic USN. Participants will use the NeuroNavAR app over a 4-week intervention. The trial will employ a mixed-methods evaluation framework:

1. *Usability and performance*: average time per AR sequence, success rate in guiding the virtual bee along object contours (e.g., percentage of completed cycles), System Usability Scale (SUS) scores from patients and therapists, and qualitative feedback on comfort and engagement;
2. *Clinical efficacy*: pre- and post-intervention assessments using the Behavioral Inattention Test (BIT) and Star Cancellation Test to quantify changes in spatial attention and functional independence.

Furthermore, building on our project’s integration of multimodal data, we plan to explore EEG and MRI biomarkers to investigate training-induced neuroplasticity and develop predictive models for personalized intervention. These comprehensive data will inform iterative refinements—such as adaptive bee movement patterns and feedback mechanisms—and lay the groundwork for larger-scale trials and eventual clinical translation.

## 6 Conclusions

This study introduces an innovative augmented reality (AR) intelligent training program, NeuroNavAR, designed to aid in the rehabilitation of stroke patients suffering from unilateral spatial neglect. The program utilizes image processing algorithms to identify and segment objects in the real world, guiding patients to follow a virtual bee pattern and enhancing engagement through real-time feedback. Experimental results indicated that this method achieved a 76.70% Intersection over Union (IoU) and an 88.51% accuracy on a test dataset of 537 indoor images, demonstrating its effectiveness in accurately recognizing and segmenting complex indoor objects. Compared to traditional rehabilitation methods, this approach offers a more intuitive, engaging, and contextually relevant training experience, which has the potential to enhance rehabilitation outcomes, subject to clinical validation.

Importantly, the foundation laid in this work sets the stage for upcoming clinical validation. Planned pilot trials will assess the system’s usability, safety, and therapeutic impact in real-world rehabilitation settings. By integrating clinical outcome measures with real-time interaction data, we aim to establish NeuroNavAR as a scalable, evidence-based digital therapeutic tool. This research represents a promising step toward accessible, personalized, and technology-driven neurorehabilitation for post-stroke cognitive impairments.

## Data Availability

The original contributions presented in the study are included in the article/Supplementary Material, further inquiries can be directed to the corresponding author.
